# A nationwide survey of the tabanid fauna of Cameroon

**DOI:** 10.1186/s13071-021-04894-0

**Published:** 2021-08-09

**Authors:** Silas L. Sevidzem, Aubin A. Koumba, Genevieve L. Yao-Acapovi, Jacques F. Mavoungou

**Affiliations:** 1 Programme Onchocercoses Field Station Laboratory, Ngaoundéré, Cameroon; 2 Département de Biologie et Ecologie Animale, Institut de Recherche en Ecologie Tropicale (IRET/CENAREST), Libreville, Gabon; 3 Laboratoire d’Ecologie Vectorielle (LEV), Libreville, Gabon; 4 Laboratoire de Biologie et Santé, UFR Biosciences, Université Félix Houphouët-Boigny, Abidjan, Côte d’Ivoire; 5 Université Des Sciences et Techniques (USTM), Franceville, Gabon

**Keywords:** Tabanids, Checklist, Abundance, Agro-ecological zone, Distribution maps, Cameroon

## Abstract

**Background:**

Tabanids are a neglected group of haematophagous dipterans despite containing 4434 species, regrouped in > 144 genera. They are mechanical vectors of important pathogens, including viruses, bacteria and protozoa of humans and domesticated and wild animals. As it is > 50 years since the publication of a preliminary nationwide record of the tabanids of Cameroon identified 84 species, updated information is needed. The aim of this study was to provide current data on the species composition, abundance and distribution of tabanids in the five main agro-ecological zones (AEZs) of Cameroon.

**Methods:**

From 2015 to 2017, a systematic entomological study using Nzi, Vavoua, Biconical and Sevi traps (*n* = 106) was conducted in 604 trapping points over 11,448 trap-days in the five main AEZs of Cameroon.

**Results:**

A total of 25,280 tabanids belonging to 25 species were collected, including eight species not previously documented in Cameroon, namely *Tabanus latipes* (1 female), *Tabanus ricardae* (1 female), *Tabanus fasciatus* (32 females and 6 males), *Haematopota pluvialis* (18 females), *Haematopota decora* (19 females and 3 males), *Haematopota nigripennis* (18 females), *Chrysops distinctipennis* (47 females and 5 males) and *Ancala fasciata* (34 females and 7 males). The distribution maps of the newly identified tabanids differed between AEZs, with most tabanids collected from the Guinean savanna. The highest apparent density of tabanids was recorded in the Sudan Savanna region, and the mean apparent densities of species with sites was statistically significantly different (Student t-test: 2.519, *df* = 24, *P* = 0.019). The highest species diversity was found in the rainforest.

**Conclusions:**

This study increased the list of tabanids recorded in Cameroon from 84 species in the preliminary record to 92 species, with most of the newly identified species occurring in the Guinea Savanna AEZ. The high diversity and abundance of tabanids in the livestock/wildlife interface areas of the rain forests and Sudan Savanna AEZs, respectively, suggest risk of mechanical transmission of pathogens. Investigations of the microbiota of tabanids in the different AEZs to define their role as disease vectors are proposed.

**Graphical abstract:**

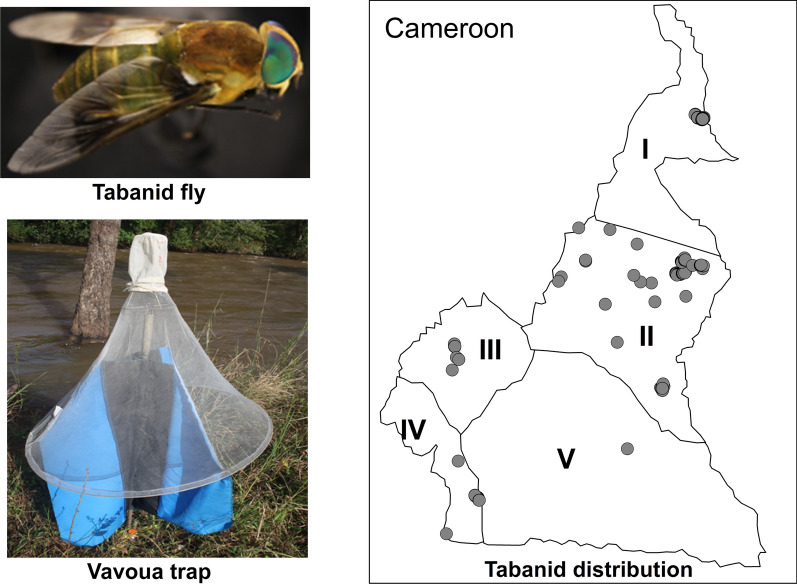

**Supplementary Information:**

The online version contains supplementary material available at 10.1186/s13071-021-04894-0.

## Background

Tabanids (Diptera, Tabanidae) form one of the largest groups of haematophagous flies with about 4434 species [1, 2] and  more than 144 genera [3–7]. These flies are well known due to three medical and veterinary important taxa, namely horse flies (*Tabanus* spp.), deer flies (*Chrysops* spp.) and clegs (*Haematopota* spp.). Tabanids inflict direct effects, such as nuisance and painful bites leading to blood loss, stress and weight loss of the affected host, and indirect effects by transmitting major pathogenic agents, including bacteria, viruses, protozoa and helminths [8–11], mechanically [12, 13]. The major blood-meal hosts of tabanids are humans, ruminants and wild animals [10, 14]. The consequence of their blood-feeding trait is the mechanical transmission of one of the most important cattle diseases in sub-Saharan Africa (SSA), namely African animal trypanosomiasis [8, 10, 15–18], in addition to the biological transmission of *Loa loa* filariasis [19, 20]. The importance of studying tabanids in relation to the mechanical transmission of pathogenic trypanosomes was emphasised at the 1948 African Conference on Tsetse and Trypanosomiasis in Brazzaville [21]. However, despite the importance of tabanids, they are a neglected subject of research [10, 22].

Tabanids of central Africa and other Ethiopian regions are either misidentified or poorly known and this applies in Cameroon. Apart from preliminary studies that reported 62 [21] and 84 [23] species respectively, there appear to have been no updates on their checklist and distribution. A survey in the Central African Republic indicated that 54 to 64 species constituted the tabanid fauna of this country [24]. The updated checklist of tabanids of the Ivory Coast reported the occurrence of 70 species [25], whereas 49 tabanid species are found in Algeria [26, 27] as confirmed in a recent update [28]. Moreover, other publications provided current information on tabanids of Kenya [29], East Africa (Uganda, Tanzania and Kenya) [30] and Gabon [18, 31, 32]. However, in Cameroon, apart from previously mentioned studies [21, 23], other reports only presented a scanty record in the Sudan savanna [33, 34]. In this context, the present study was aimed to update information on the diversity, abundance and distribution of tabanids in the main AEZs of Cameroon.

## Methods

### Description of collection sites

**Fig. 1:**
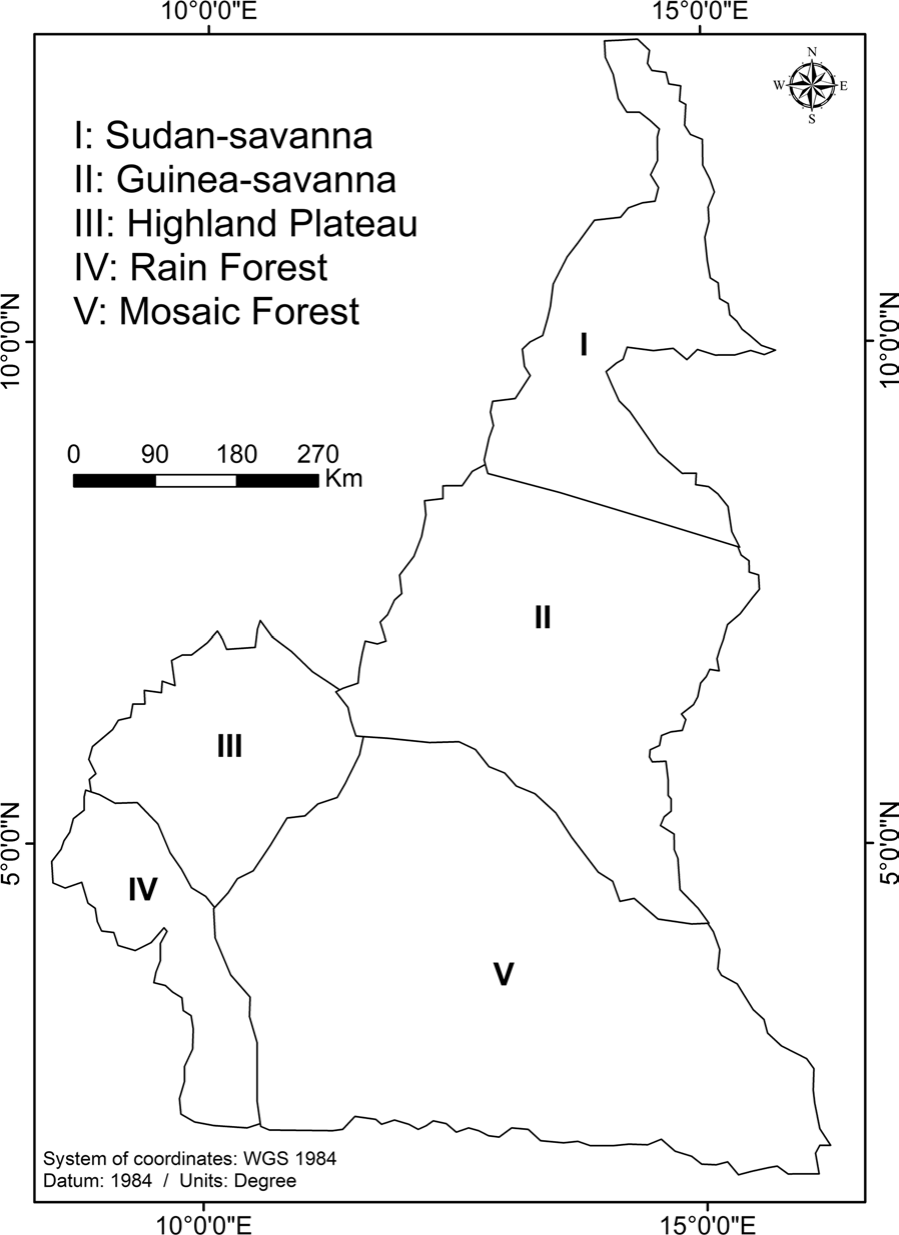
Map showing the different agro-ecological zones of Cameroon

The study zones consisted of the five main AEZs of Cameroon (Fig. 1). The geographical coordinates and climatic characteristics of the different AEZs are described in Table 1.

**Table 1:** The surveyed agro-ecological zones in Cameroon with their geographical coordinates and climatic characteristics

SN	Agro-ecological zone	Study region	GPS coordinates	Altitude (m a.s.l.)	Climatic conditions of fly collection sites
I	Sudan Savanna	Far North	10°775’N	304	Sudano-sahelian climate with two seasons (rainy and dry). Average monthly temperature is 28 °C. Rainfall is 400–1200 mm/year
			14°917’E		
		North	7°77796’N	545	
			14°929’E		
II	Guinea Savanna	Adamawa	7°00334’N	1000	Sudanese climate type with average monthly temperature of 20–26 °C. Rainfall is 1500 mm/year
			13°01’E		
III	Highland Plateau	North-west	5°92523’N	900	Cold climate with mean monthly temperature of 19 °C. Rainfall is 1500–2000 mm/year
			10°009’E		
IV	Rainforest	Littoral	3°23333’N	35	Very humid, hot and variable equatorial climate with average monthly temperature between 22 and 29 °C. Rainfall is 2500–4000 mm/year
			9°567’E		
V	Mosaic forest	East	6°23333’N	890	Guinean climate type (hot and humid). Rainfall is 1500–2000 mm/year
			13°25’E		

SN, Site number

### Entomological field surveys

Tabanid trapping was conducted simultaneously in all five AEZs for 3 years (2015–2017) using different trap types, including Nzi, Vavoua, Biconical and Sevi traps. Nzi, Vavoua and Biconical traps have been used to catch tabanids in previous studies carried out in Cameroon [35, 36], whereas the Sevi trap is a modification of the malaise (René Malaise) and Canopy traps [37]. It was designed by the first author (SL Sevidzem) in Ngaoundere, tested by the Special Mission for Tsetse flies Eradication (MSEG) team in the tsetse fly-infested region of North Cameroon and confirmed as a trap for tabanids [33]. A description of the Sevi trap is provided in Additional file 1: Text 1. The same number of traps and types were not deployed in the five sampled AEZs due to differences in topography, limited number of traps/types and limitations in the number of personnel to monitor the traps. The geolocation of trapping points was conducted using a Global Positioning System (GPS) handset (GPS eTrex®; Garmin [Europe] Ltd., Southampton, UK). The trapping effort, defined as the number of traps multiplied by the number of trapping days (trap-days), was recorded. The present study was conducted within 108 days using 106 traps, resulting in a trapping effort of 11,448 trap-days.

### Sudan Savanna of the Far North region

The study in the Sudan Savanna of the Far North region was carried out using 20 Nzi traps [38] in the following sites: Kalang, Kainide, Diddel tanne, Doulam and Yanga. The traps were set in potential tabanid breeding areas, such as in marshy areas around livestock drinking points, in open grass savanna that represented livestock grazing spots and in gallery forests. Trapping was conducted during 3 consecutive days per month throughout the study period. Traps were set and activated in the morning (08:00 h) and cages emptied in the evening (06:00 h).

### Sudan Savanna in the North region

Trapping in the Sudan Savanna in the North region was carried out using a total of 39 traps (25 Vavoua [39], 10 Biconical [40], 2 Nzi [38], 2 Sevi [33]) in three sites: Mbele, Zone 27 and Zone 26. Trapping sites were mostly beside the Game Reserve, beside the river and at livestock grazing spots. Traps were activated in the morning (08:00 h) and their cages emptied every evening (06:00 h) for 3 consecutive days per month throughout the study period.

### Rainforest of the Sanaga-Maritime

In the rainforest of Sanaga-Maritime, trapping sites consisted of the abattoir (open forest), palm oil plantation (open forest with mainly tall palm trees and > 50 N’Dama cattle) and around the game reserve (humid and closed forest with tall canopy trees). A total of 18 traps were used: Biconical (*n* = 6), Vavoua (*n* = 6) and Nzi (*n* = 6). The traps were set in the morning (06:00 h) and emptied every evening (06:00 h) for 3 consecutive days per month throughout the study period.

### Guinea Savanna of the Adamawa plateau

The trapping sites in the Guinea Savanna zone consisted of Velambai (Lake Djalingo with open grass savanna), Mbidjoro (open grass savanna, forest–savanna mosaic) and Vina du Sud (Vina River with gallery forest). The trapping points were in villages with intensive cattle breeding activities. As in the rainforest of the Sanaga, a total of 18 traps were used: Biconical (*n* = 6), Vavoua (*n* = 6) and Nzi (*n* = 6). The traps were set in the morning (07:00 h) and their cages emptied every evening (06:00 h) for 3 consecutive days per month throughout the study period.

### Mosaic forest in the East region

The survey in the mosaic forest in the East region was carried out using a total of six traps: Nzi (*n* = 3) and Vavoua (*n* = 3) traps. The traps were set in three environments: gallery forest, overnight cattle corrals and river banks (watering point for cattle). These three biotopes were identified in four sites: Minali, Oudou, Camp Général and Gabong of the Société de Développement et d'Exploitation des Productions Animales (SODEPA). The trapping points were all on the SODEPA ranch which is a public structure involved in intensive cattle production activities. The trapping cages were emptied after 24 h on 3 consecutive days per month throughout the study period.

### Highland Plateau of the North-west region

In the Highland Plateau of the North-west region, five Biconical traps were set at livestock drinking points and grazing areas in the following sites: Bali Top Quarters, Saphery, Babah, Njinki, Tchaboutchou, Munam and Ntchuobo. Flies were collected from the traps each day before nightfall (06:00 h) for 3 consecutive days per month throughout the study period.

### Fly identification

All specimens were conserved in ethanol [41] and identified using a stereo microscope (Carl Zeiss™ STEMI 2000-C; Carl Zeis Microscopy GmbH, Jena, Germany). The identification of tabanid flies was made following published morphological identification keys [42–44]. The species recorded by Ovazza et al. [23] and used in this present study were rewritten following Chainey and Oldroyd [45].

### Sex determination

The sex of newly identified tabanid specimens was determined using published criteria [43], where females naturally possess a larger inter-ocular width than their male counterparts.

### Determination of abundance

The abundance of trapped tabanids was translated as their apparent density per trap, reported as the number of flies per trap per day [18] as follows:

$${\text{ADT}} = \frac{{{\text{NTC}}}}{{{\text{NT }} \times {\text{ND}}}}$$

where ADT is the apparent density per trap, NTC is the number of tabanids captured, NT is the number of traps and ND is the number of trapping days.

### Statistical analyses

Field survey records were completed in Access 2013 (Microsoft Corp., Redmond, WA, USA) databases and joined to the location shape files produced from gpx flies from GPS handsets in ArcMap™ version 10.1 Geographic Information System (GIS) software (Environmental Systems Research Institute, West Redlands, CA, USA). Data were analysed using the JASP 0.13.0.0 statistical software [46]. The Student t-test was used to compare the mean ADT of tabanid species with sites of the different AEZs, with significance set at *P* < 0.05.

## Results

### Distribution of tabanids in the study sites

**Fig. 2:**
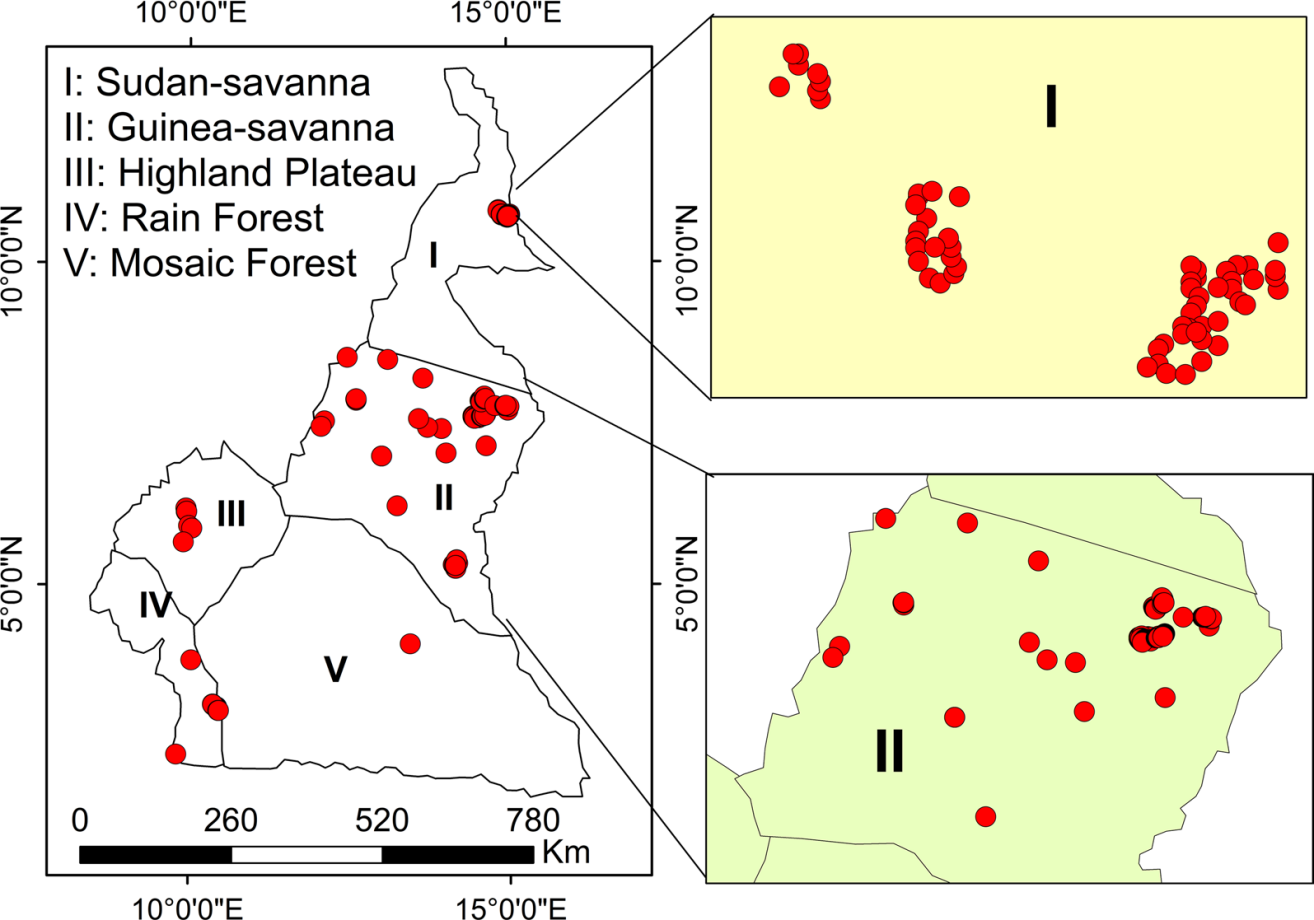
Map showing the trapping points in the study zones of Cameroon

The present study was conducted from 2015 to 2017 at 25 sites in the five major AEZs of Cameroon, with a total of 604 trapping points (Fig. 2). A total of 25,280 tabanid specimens were collected during the study period, with 25 identified species regrouped under five genera (*Tabanus*, *Chrysops*, *Haematopota*, *Ancala* and *Atylotus*) which were in turn members of three tribes: Tabanini, Chrysopini and Haematopotini. At the genus level, members of genera *Haematopota*, *Ancala*, *Chrysops* and *Tabanus* were encountered more often in the mosaic forest/rainforest than in the savanna, whereas members of genus *Atylotus* were only encountered in the savanna and never trapped in the forest. *Chrysops longicornis* was the only *Chrysops* species that was identified in both forest and savanna collections, with the other *Chrysops* either restricted to the forest (*C. silacea*, *C. dimidiata*, *C. funebris*) or to the savanna (*C. distinctipennis*). *Haematopota decora* and *Ancala fasciata* were found in both forest and savanna collections, whereas *Atylotus agrestis* was restricted to sites in the Guinea Savanna and Sudan Savanna. Only *Tabanus taeniola* was caught in all the study sites (Additional file 2: Table S1).

### The abundance of tabanids with study sites

The species of tabanids caught in the different sites recorded highest abundance in some sites as compared to others. Highest ADT occurred at Diddel Tanne in the Sudan savanna of the Far North region, with such dominance caused by *Atylotus agrestis* (136.25 flies per trap per day (f/t/d)). Lowest ADT was recorded in most sites in the plateau highland of the North West region (Additional file 3: Table S2). However, the mean ADTs of tabanid species with sites was statistically significantly different (Student t-test: 2.519, *df* = 24, *P* = 0.019).

### Species diversity in the study sites

**Fig. 3:**
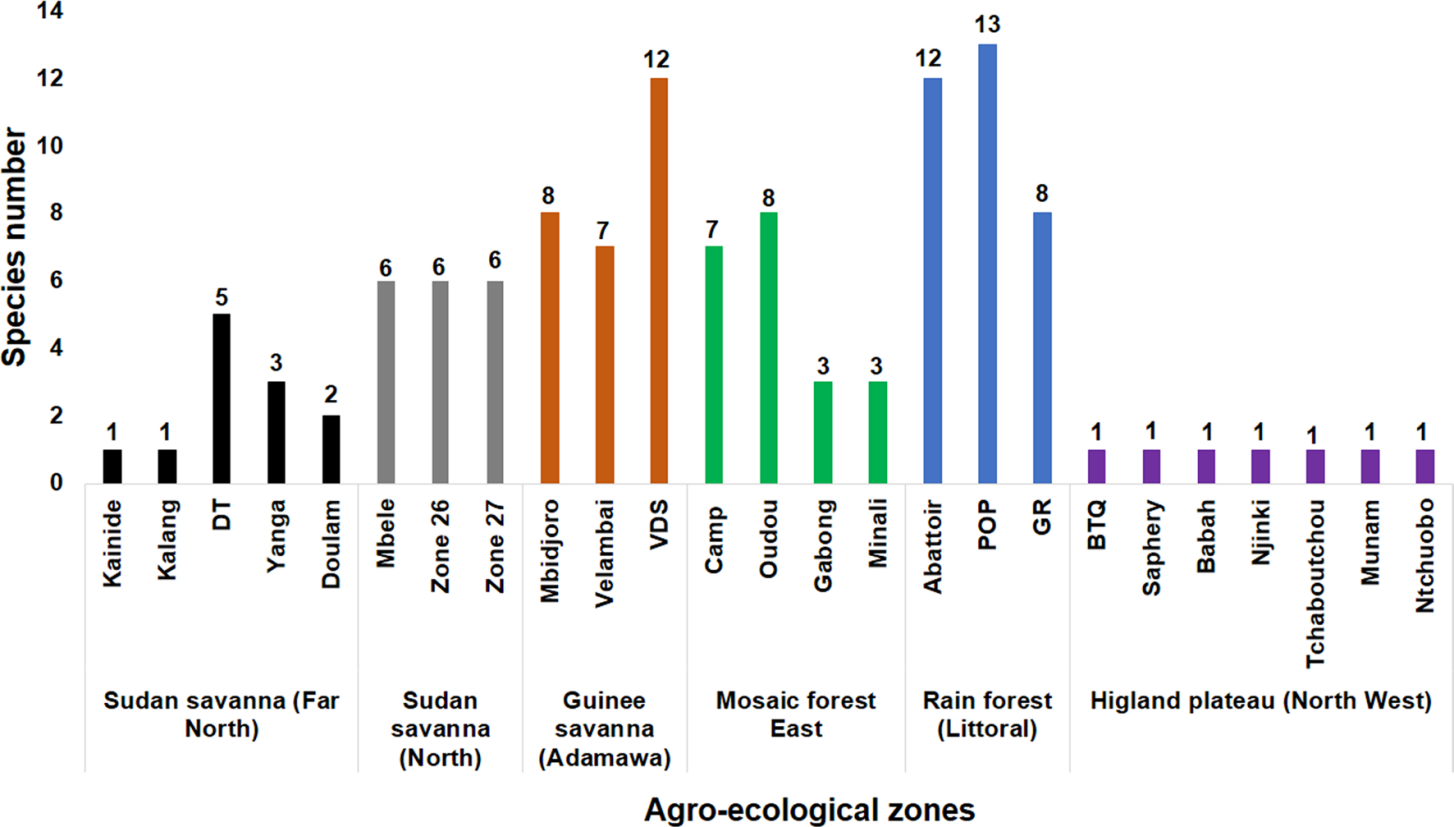
Number of species of tabanids collected in the study sites and agro-ecological zones (2015–2017).* DT* Diddel tanne,* VDS* Vina du Sud, *POP* palm oil plantation,* GR* game reserve,* BTQ* Bali Top Quarters

The site with highest species diversity was the palm oil plantation (13 species) located in Mouanko around the Douala-Edea Zone (DEZ) in the rainforest, followed by Vina du Sud (12 species), a riverine-gallery forest–savanna mosaic site. Sites at which the least number of species (1 species) was identified in collections were: Kainide and Kalang sites (savanna of the Far North region) and in the Bali Top Quarters (BTQ), Saphery, Babah, Njinki, Tchaboutchou, Munam and Ntchuobo sites in the Highland Plateau of the North-west region (Fig. 3).

### Checklist of the Tabanidae of Cameroon

Information on the nationwide zoogeographical distribution of tabanids in Cameroon was last updated over half a century ago, with 84 species reported to occur in Cameroon [23]. Taxonomically, tabanids from the reviewed data sets (84 species) plus those from the present study (25 species, including 8 new species and 17 species reported earlier [23]) belong to three subfamilies: Tabaninae, Pagoniinae and Chrysopinae. The species are placed in 15 genera (92 species): *Hippocentrum* (2 spp.), *Haematopota* (19 spp.), *Ancala* (3 spp.), *Euancala* (1 sp.), *Atylotus* (3 spp.), *Philoliche* (3 spp.), *Chrysops* (9 spp.), *Sphecodemyia* (1 sp.), *Thriambeutes* (1 sp.), *Jashinea* (1 sp.), *Tabanocella* (3 spp.), *Tabanus* (43 spp.), *Thaumastocera* (1 sp.), *Hybromitra* (1 sp.) and *Mesomyia* (1 sp.). These genera belong to six tribes: Bouvieromyini, Rhinomyzini, Chrysopini, Philolichini, Tabanini and Haematopotini (Additional file 4: Table S3).

The recent surveys (2015–2017) led to the identification of eight species that were not present in the list of tabanids of the preliminary survey and increased the list of tabanids occurring in Cameroon to 92. The collection sites as well as the distribution maps of the eight recently identified species are described in the following sections.

#### *Tabanus latipes*

**Fig. 4:**
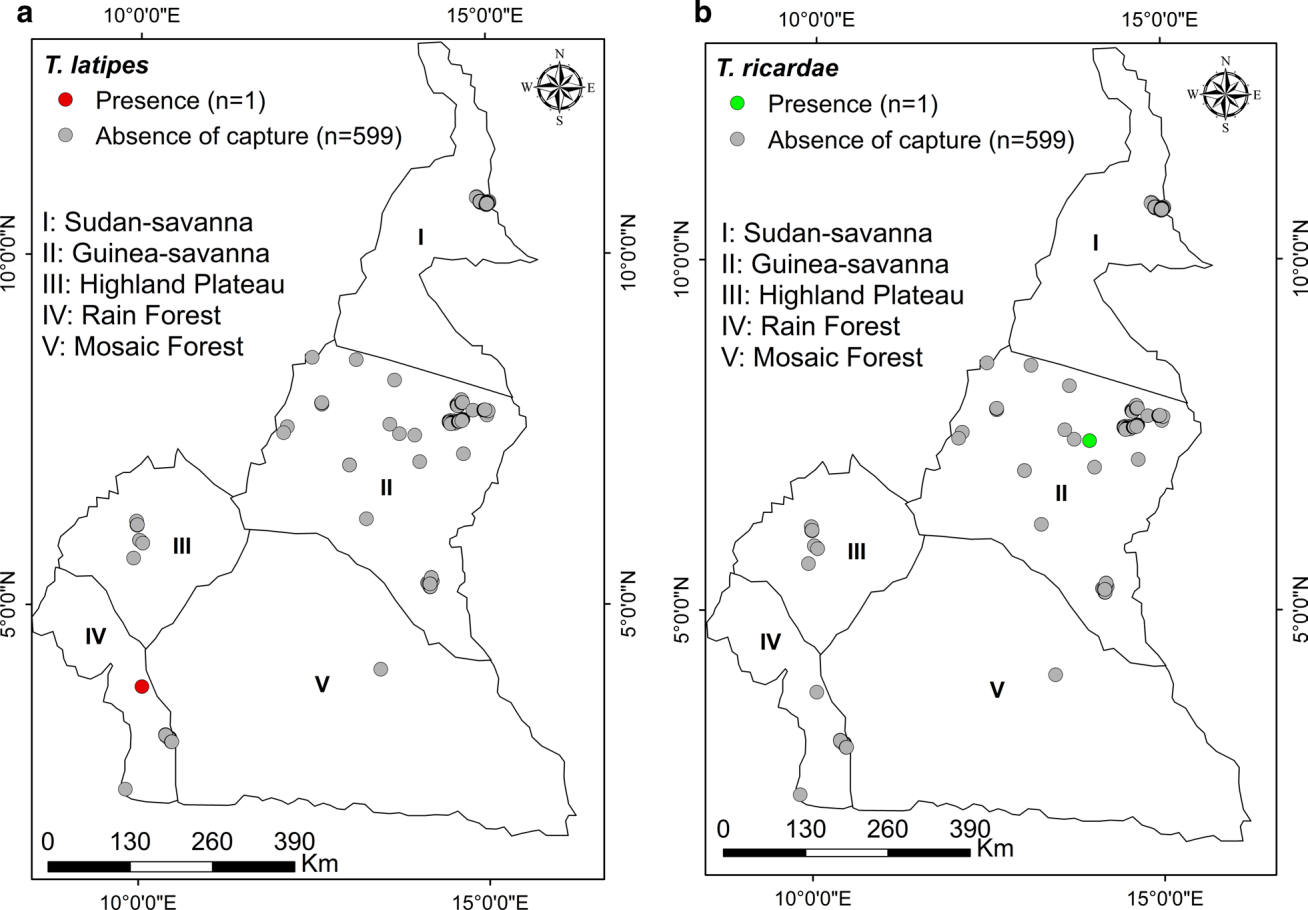
Distribution map of *Tabanus latipes* (**a**) and *Tabanus ricardae* (**b**)

Only one female of this species was caught using a Nzi trap set in the marshy land of the abattoir (3°83333′N, 10°05′E) in Mouanko in the rainforest of the DEZ of Cameroon (Fig. 4a).

#### *Tabanus ricardae*

Only one female of this species was caught by a Nzi trap set in the marshy cattle grazing field around Lake Djalingo (7°42667′N, 13°945′E) of Velambai in the Sudan Savanna (Fig. 4b).

#### *Tabanus fasciatus*

**Fig. 5:**
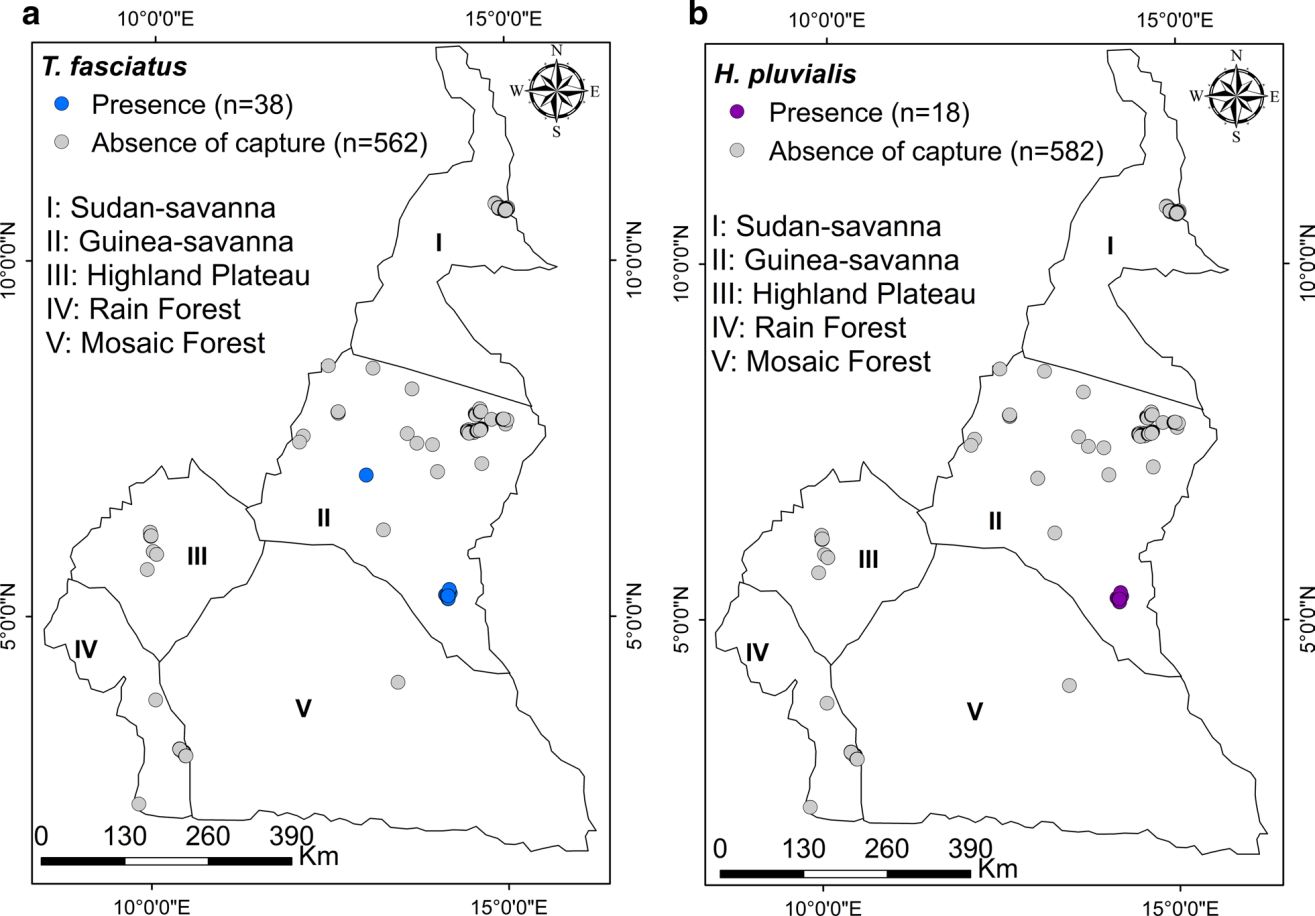
Distribution map of *Tabanus fasciatus* (**a**) and *Haematopota pluvialis* (**b**)

Individuals (32 females and 6 males) of this species were caught with a Nzi trap set in the gallery forest (7°00,334′N, 13°01′E) of the Sudan Savanna of Adamawa, in the Camp site and Oudou, Gabong and Minali sites of the SODEPA ranch (4°0833′N, 13°45′E) of Ndokayo in the mosaic savanna (Fig. 5a).

#### *Haematopota pluvialis*

Only females (*n* = 18) of this species were caught with Nzi traps set in the mosaic forest (5°33972′N, 14°188′E) precisely in Gabong of the East region (Fig. 5b).

#### *Haematopota decora*

**Fig. 6:**
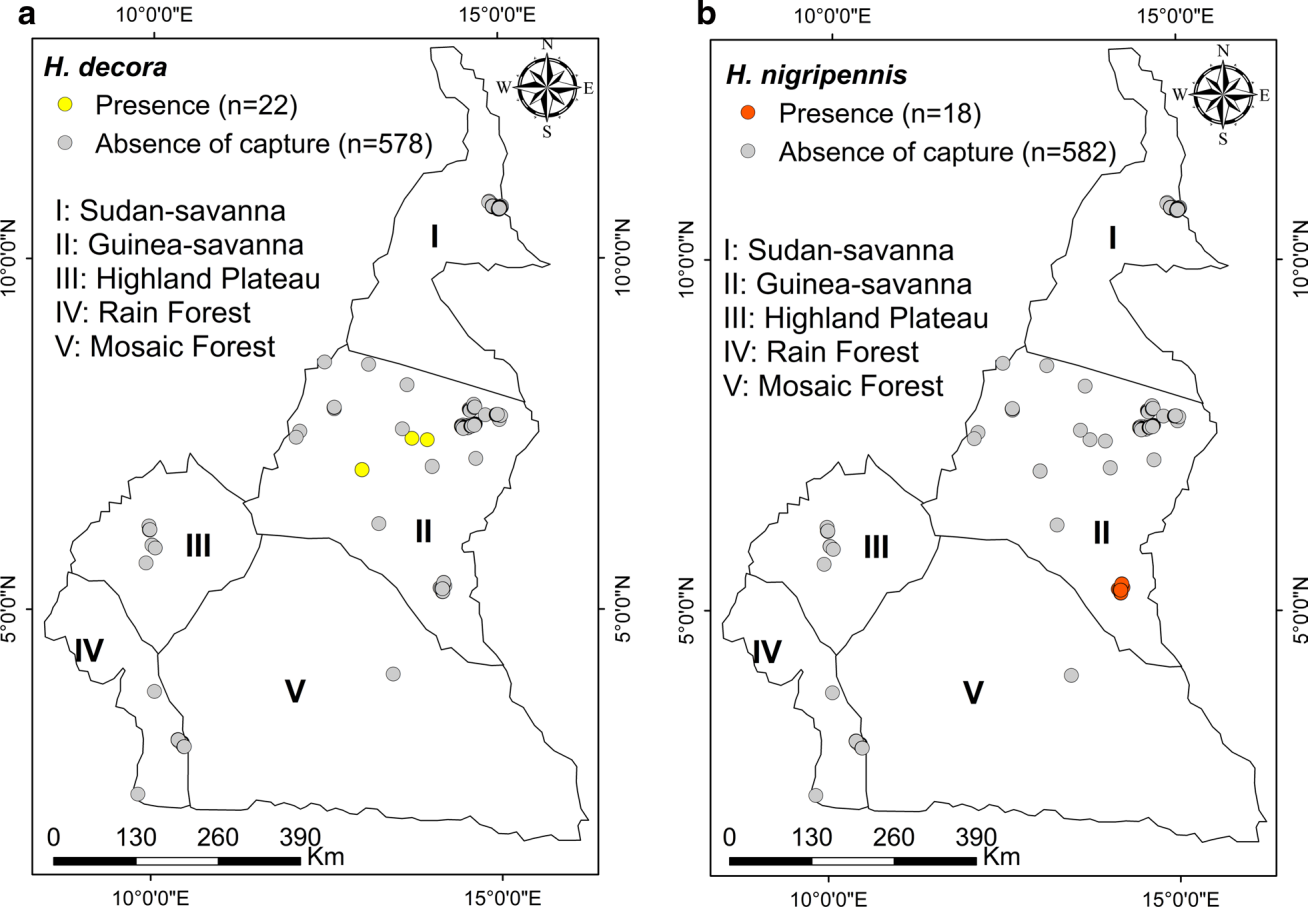
Distribution map of *Haematopota decora* (**a**) and *Haematopota nigripennis* (**b**)

Members of this species (19 females and 3 males) were caugh with Nzi and Vavoua traps in the savanna of Adamawa, more precisely in Galim (7°00334′N, 13°01′E), Velambai (7°42667′N, 13°945′E) and Mbidjoro (7°44778′N, 13°726′E) (Fig. 6a).

#### *Haematopota nigripennis*

Only females (*n* = 18) of this species were caught with Nzi traps set in Gabong of the mosaic savanna of SODEPA ranch (4°0833′N, 13°45′E) (Fig. 6b).

#### *Chrysops distinctipennis*

**Fig. 7:**
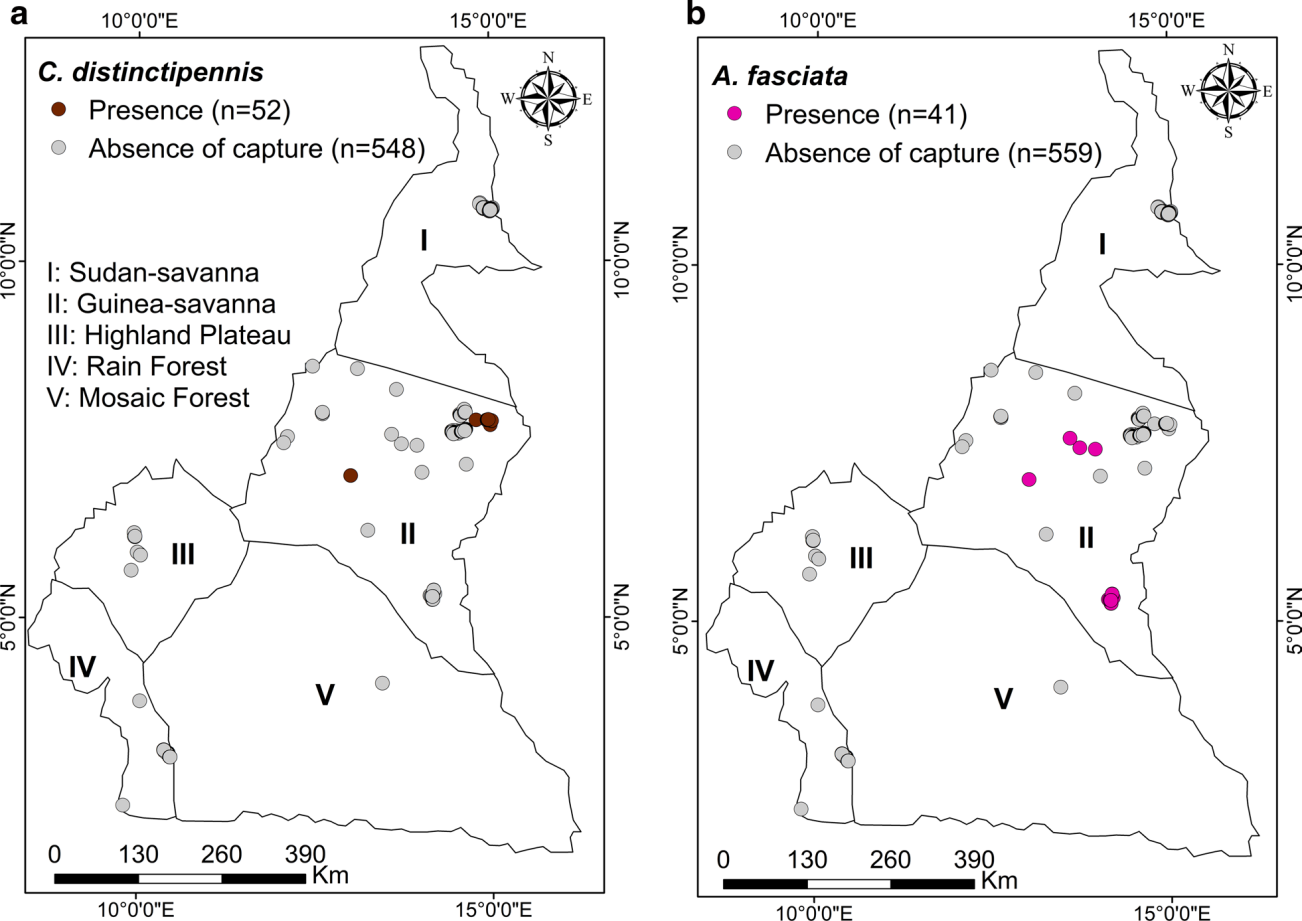
Distribution map of *Chrysops distinctipennis* (**a**) and *Ancala fasciata* (**b**)

This species was caught (47 females and 5 males) using Nzi traps set in the Guinea Savanna of Adamawa (7°00335′N, 13°01′E) and in the North and Far North regions of the Sudan Savanna (7°77,115′N, 14°983′E) (Fig. 7a).

#### *Ancala fasciata*

This species was caught (34 females and seven males) using Nzi traps set in some sites in the rainforest (palm oil plantation [3°23333′N, 9°567′E) and abattoir [3°83333′N, 10°05′E]) in the DEZ, the Guinea Savanna (7°00335′N, 13°01′E) and the mosaic savanna (5°25694′N, 14°155′E) (Fig. 7b).

## Discussion

These surveys, conducted from 2015 to 2017, led to the identification of 25 tabanid species, much fewer than the 84 species recorded in the preliminary study of 1970 [23]. Of these 25 species, 17 were already known and the other eight had not been previously recorded in Cameroon, including *T. latipes*, *T. ricardae*, *T. fasciatus*, *H. pluvialis*, *H. decora*, *H. nigripennis*, *C. distinctipennis* and *A. fasciata*. The new species records increase the list of tabanids identified in Cameroon to 92 species, which is higher than that reported in other countries within the Afro-tropical region, including the Central African Republic [24], Ivory Coast [25], Algeria [26–28], Kenya [29, 30], Zambia [17], South Africa [17], Nigeria [47], Uganda and Tanzania [30], Congo [48], Liberia [49] and Gabon [18, 31, 32]. The higher number of species in the preliminary survey compared to this present study could be due to several factors, including the limited number of sampled sites (*n* = 25) in the different AEZs in the current study compared to several (> 25) sites in the preliminary study, the use of sweep nets in the preliminary study compared to tsetse traps in the present study and ecological changes that have influenced fly distribution and densities.

The higher number of tabanid species in Cameroon compared to that of the other countries of the Afro-geographical region likely reflects the presence of more diverse AEZs contributing to the successful development and survival of several species of tabanids. The newly recorded tabanid species of Cameroon are present in neighbouring countries of the Central and West African subregions [17, 18, 25, 31, 47]. A review on the genus *Chrysops* of Africa with a focus on Cameroon [20] mentioned only the occurrence of *C. silacea* and *C. dimidiata*, whereas our survey revealed five species, notably *C. distinctipennis*, *C. longicornis*, *C. funebris*, *C. silacea* and *C. dimidiata*. One possible reason for the previous scanty record of the species of this genus [20] may be that the earlier entomological surveys were limited to the forest regions of Cameroon, where *C. silacea* and *C. dimidiata* are omnipresent. Interestingly, the 1955 report on the presence of five species of *Chrysops* [21], notably *C. dimidiata*, *C. silacea*, *C. funebris*, *C. longicornis* and *C. stigmaticalis*, was conducted in the same study areas as those in the review on the genus *Chrysops* [20]. However, neither of these reports identified *C. distinctipennis*, a species recorded in the Guinean Savanna ecozones of Cameroon in our current study.

From the eight newly recorded tabanid species of Cameroon, only *H. pluvialis* is a Palaearctic species. *Haematopota pluvialis* inhabits various types of biotopes but is particularly very common near water and on foggy biotopes where the larvae live [51, 52]. *Haematopota pluvialis* is the most common tabanid species in Europe [51, 53]. Females actively attack humans and animals, mostly in the evening and in cloudy and showery weather [51]. The flight activity of *H. pluvialis* mostly depends on the relative humidity, but air temperature also had a great effect [52]. The flight period for this species mostly extends from late May to the beginning of October in some parts of Europe, with maximal flight activity occurring in July and August [51, 52].

*Tabanus taeniola* was the most abundant species in the forest and savanna, likely reflecting the environmental conditions favourable to this species, with similar favourable conditions frequently reported in the collections of other authors in the Afro-geographical zone [15, 18, 33, 50, 54]. Further, the highest tabanid mean apparent density was recorded in the Sudan Savanna AEZ, a finding similar to that presented in the 2020 report of the MSEG [34, 55], indicating the apparent absence of tsetse flies in this region and the high apparent densities of tabanids. This highest abundance of tabanids in sites of the Far North region of the country is likely due to this area being a major cattle-rearing region of Cameroon [56, 57] as well as containing the Waza Park that harbours diverse and high numbers of domestic and wild animals that can serve as blood-meal hosts for this fly group. The presence of cattle and wild animal hosts, plus the conducive environmental conditions of this region, presumably favours the development and survival of tabanids.

The highest tabanid species diversity was recorded in the rainforest of the DEZ in comparison to the other AEZs. The highest number of species (*n* = 13) was also recorded in the rainforest compared with the other sampled AEZs. One likely factor accounting for this high abundance and diversity could be the favourable environmental conditions of this AEZ for several species of tabanids and because it was the least anthropized of the sampling sites, with wild animals and N’Dama cattle serving as blood-meal hosts and providing a suitable environment for tabanid development and survival [31]. The rainforest sites consisted of tall palm trees equally interspaced to provide high luminosity and canopy trees for shelter, rivers and abundant marshy land for breeding; the presence of palm trees has been reported to harbour high numbers of different species of tabanids [23]. The 1955 study in forest areas of South Cameroon indicated the abundance of breeding sites for female tabanids that favoured their proliferation in this zone [21].

## Conclusions

The present study registered 25,280 tabanid specimens that were regrouped under 25 species. Of the 25 species recorded, eight had not been included in the preliminary list of Tabanidae of Cameroon previously published [23], notably *T. latipes*, *T. ricardae*, *T. fasciatus*, *H. pluvialis*, *H. decora*, *H. nigripennis*, *C. distinctipennis* and *A. fasciata*. Identification of these novel species has increased the number of Tabanidae occurring in the country from 84 to 92. The newly identified species were mostly found in the Guinean Savanna. The highest Tabanidae apparent density was recorded in the Sudan Savanna region whereas the highest species diversity was noted in the rainforest of the DEZ. The high diversity and abundance of Tabanidae in the livestock/wildlife interface areas of the rainforest and Sudan Savanna AEZs, respectively, strongly suggests a risk for the mechanical transmission of dangerous pathogens. Future investigations on this group focussed on studying the microbiota they harbour are proposed, with the aim to establish their potential epizootiological role in the transmission of diseases in the different AEZs.

## Supplementary Information

**Additional file 1: Text 1.** Detail presentation of the Sevi trap.

**Additional file 2: Table S1.** Species distribution of tabanids in different sites and bioclimatic zones of Cameroon (2015–2017). Asteriks (*) indicate the presence of species.

**Additional file 3: Table S2.** The abundance of tabanids in the different sites of the different bioclimatic zones (2015–2017).

**Additional file 4: Table S3.** Checklist, classification and collection sites of tabanids of Cameroon reported between 1970 and 2017. Species indicated with an asterisk (*) are those identified between 2015 and 2017, which were not found in the list of tabanids of Cameroon reported by Ovazza et al. [23].

## Data Availability

All data generated or analysed during this study are included in this published article and its supplementary information files. The description of the Sevi trap that was used in this study for the first time is provided within Additional file 1: Text 1.

## Abbreviations

CARPA: Center for Support to Research and Pastoralism
IRAD: Agricultural Research Institute for Development
MINEPIA: Ministry of Livestock Fisheries and Animal Industries
MSEG: Special Mission for Tsetse flies Eradication
