# Supplementary material for: A nationwide survey of the tabanid fauna of Cameroon
Source: Parasit Vectors. 2021 Aug 9;14:392. doi: 10.1186/s13071-021-04894-0 (PMC8351441; doi:10.1186/s13071-021-04894-0)
Supplement: Supplementary file 1 — Additional file 1: Text 1. Detail presentation of the Sevi trap. [file 13071_2021_4894_MOESM1_ESM.docx]

**Additional file 1: Text 1**. Detail presentation of the Sevi trap

This trap is a modification of the malaise (René Malaise) and Canopy traps [37]. It was constructed by Sevidzem Silas Lendzele in 2015 in Ngaoundere and named it Sevi trap (Fig. 1). The aim of making the Sevi trap was to control hematophagous flies such as tabanids, glossines and stomoxyines in the major cattle rearing regions of Cameroon in collaboration with the special mission for tsetse flies eradication (MSEG). The trap consisted of a lower compartment made up of a 1 m x 0.5 m sky blue cloth (b) material seamed to a 5 cm x 0.5 m mosquito net (a) upper compartment. Four nylon ropes (0.6 m each) (f) were used to tension the cloth and keep it upright. A 1.5 m central iron rod (c) was fixed to the cone (anti-return device) (e) to keep the whole trap upright. A mosquito netted collection cage (5 cm x 15c m) (d) was tied to the cone with a rope. The trap functions thanks to its attractive blue color. The attracted flies enter the trap through the 180 cm base and faced with a dark lower core, they move towards the light mosquito net zone where they are trapped in the cage. The schematic diagram (Fig. 2) was the modified version, but the photo (Fig.1) shows the original version with difference at the level of the length of the blue material where it was previously 0.75 m long but was later modified to 0.5 m. The first field trial of the modified version in North Cameroon by the MSEG team led to the collection of stomoxyines, glossines and tabanids with apparent densities: *S. n. niger* (2.5 (flies per trap per day (f/t/d)), *S. calcitrans* (1 f/t/d), *S. n. bilineatus* (0.5 f/t/d), *S. sitiens* (0.5f/t/d), *G. tachinoides* (0.5 f/t/d), *T. taeniola* (1.5 f/t/d) and *C. longicornis* (0.5 f/t/d) [33]. From the results of the first trial it was suggested that this trap could be improved to catch tabanids, stomoxyines and glossines.


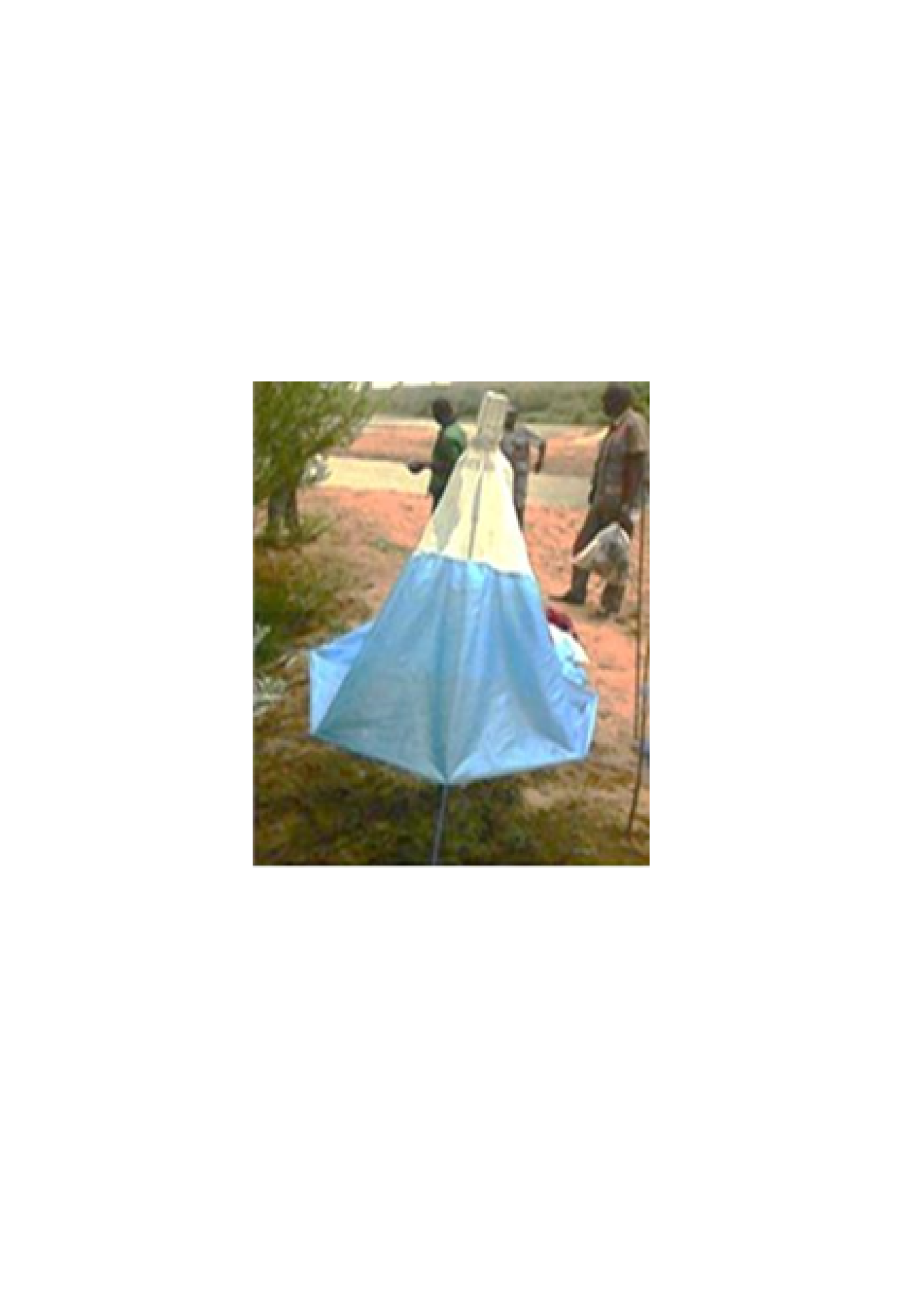

**Fig. 1** Original sevi trap.


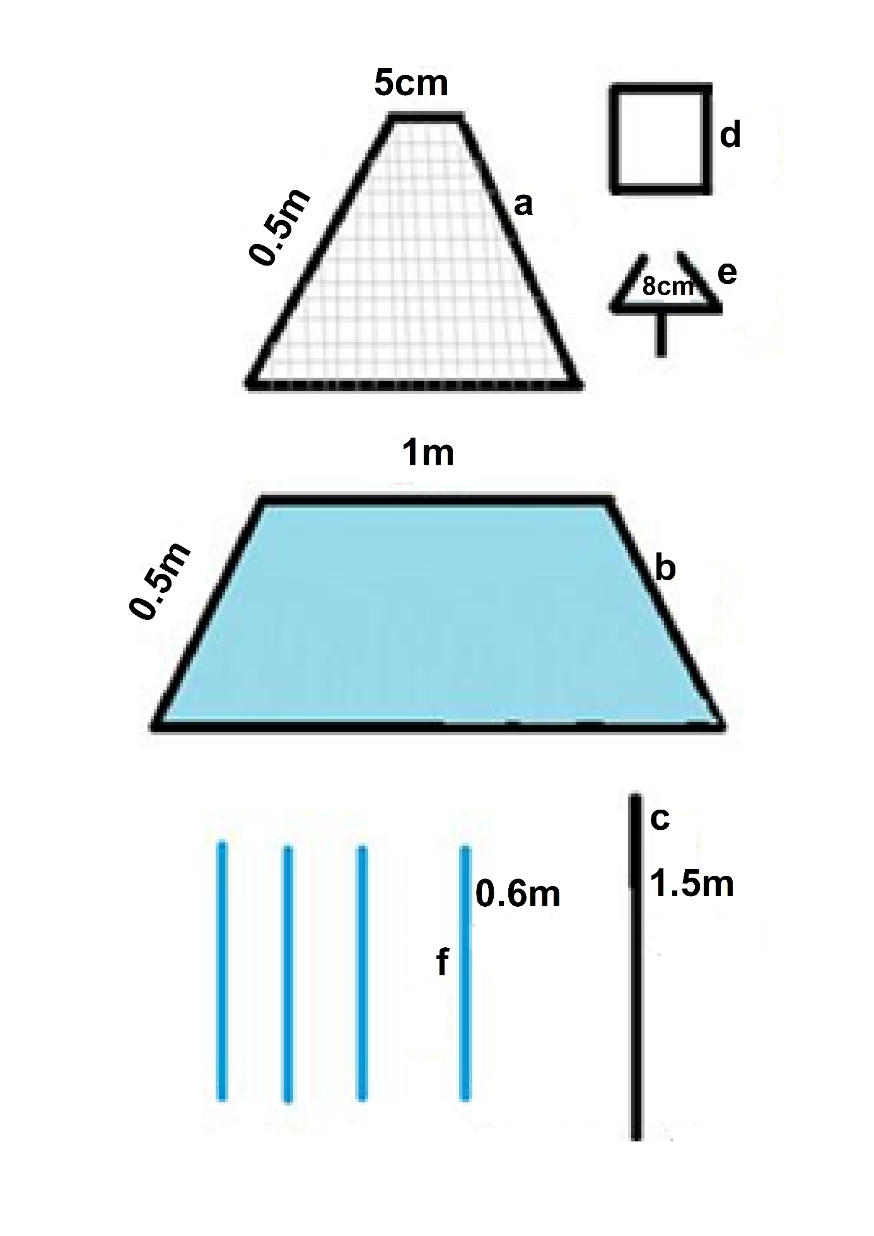


**Fig.2** Schematic presentation of the Sevi trap.
