# Supplementary material for: A nationwide survey of the tabanid fauna of Cameroon
Source: Parasit Vectors. 2021 Aug 9;14:392. doi: 10.1186/s13071-021-04894-0 (PMC8351441; doi:10.1186/s13071-021-04894-0)
Supplement: Supplementary file 4 — Additional file 4: Table S3. Checklist, classification and collection sites of tabanids of Cameroon reported between 1970 and 2017. Species indicated with an asterisk (*) are those identified between 2015 and 2017, which were not found in the list of tabanids of Cameroon reported by Ovazza et al. [23]. [file 13071_2021_4894_MOESM4_ESM.docx]

**Additional file 4: Table S3.** Checklist, classification and collection sites of tabanids of Cameroon reported between 1970 and 2017. The species in asterisk (*) are those identified between 2015 - 2017 that were not found in the list of tabanids of Cameroon reported by Ovazza et al. [23].

| **Subfamily** | **Tribe** | **Genus** | **Species** | **Collection sites** |
| --- | --- | --- | --- | --- |
| Tabaninae | Haematopotini | *Hippocentrum* | *H. strigipenne* | It was found at the slope of Mount Cameroon with the name *H. concise.* Also signalled in Metchum, Wum, Oku-forest, Yaounde, Bertoua and Maroua. All the other localities are situated in the forest and post forested zones. Thus, localities in Cameroon do not show the limit of the extension of this species. Itard et al. [24] found it in the Central African Republic (C.A.R.). According to Ovazza et al. [23], the presence of *H. strigipenne* in Cameroon was due to the presence of palm oil trees, soil in forest, galleries and savanna forest relics. The mentioned authors testified that this species was present throughout the year |
|  |  |  | *H. versicolor* | This species seemed to be rare in Cameroon. It was cited in unprecise localities by Oldroyd [58] with single indication in Maroua in the dry savanna |
|  |  | *Haematopota* | *H. griseicoxa* | As propounded by Oldroyd [58], the genus was very homogenous with diagnostic characteristics. The distribution of this genus was not only limited to the Ethiopian region but way beyond. *H. griseicoxa* can be found in Kumba, Garoua Boulai and in the Soudano-sahelian zone of Ngaoundere [23]. It is a forest species |
|  |  |  | *H. ciliatipes* | It was apparently rare and can be found in Yaounde and along the Yoko/Deng Deng road in the Lom and Djerem division (East). Mostly caught in gallery forests [23] |
|  |  |  | *H. inornata* | Its type-locality was Wum, but recent prospections revealed their presence in the Guinean-savanna of Cameroon [23] |
|  |  |  | *H. germaini* | It was caught in the Oku forest. Mostly infest gallery forests [23] |
|  |  |  | *H. rufula* | This species was identified in Cameroon for the first time by Oldroyd [58] in Galim, a Guinee-sahelian zone of the Adamawa Plateau [23] |
|  |  |  | *H. dukei* | It was caught in mount Bamboutous and two females in Dschang at an altitude of 1600 m by Ovazza et al. [23] |
|  |  |  | *H. crevei* | It was collected in Mamfe in the rainforest of the South west region of Cameroon [23] |
|  |  |  | *H. partifascia* | It was signalled in Adamawa, Banyo and Tibati. All its biotopes were located in the Guinee-savanna [23] |
|  |  |  | *H. barombi* | This species is frequent in Cameroon. It is found in forest and forest relics of mountainous regions and in transition zones, typical of Kumba [23] |
|  |  |  | *H. guineensis* | This species occurred in all forest regions, some already signalled in Meme, upper Nyong, Kribi, Mbalmayo and Bamboutous [23] |
|  |  |  | **H. pluvialis* | This species was caught in the mosaic-forest precisely in the Gabong site of East Cameroon |
|  |  |  | *H. abyssinica* | This species was first identified in Bui and along river Faro [23] |
|  |  |  | *H. ochracea* | This species was identified in Ayo Nyong |
|  |  |  | *H. patellicorne* | This species was described in the Guinee-savanna areas [23] |
|  |  |  | *H. okui* | This species was caught in the Oku forest [23] |
|  |  |  | *H. heptagramana* | This species was only known by the holotype which came from Soppo in Mt. Cameroon [23] |
|  |  |  | *H. laiessens* | This species was caught in Mamfe and Oku in humid forest areas [23] |
|  |  |  | **H. negripennis* | This species was recently caught in the mosaic-forest precisely in the Gabong site of East Cameroon |
|  |  |  | **H. decora* | This species was identified in the savanna of Ngaoundere; precisely in Galim, Velambai and Mbidjoro |
|  | Tabanini | *Ancala* | ****A. fasciata* | This species was found in dense forests of Gabon and Congo Brazzaville. It seemed not to exist in Cameroon during the study of [23] |
|  |  |  | *A. fasciata nilotica* | This species existed in the northern savannas and in forested areas such as: Upper Sanaga, Nyong and Mfoumou, Lom and Djerem, Logone and Chari [23] |
|  |  | *Euancala* | *A. latipes* | This species was identified in Tchad and north Nigeria. It is found in all forest and savanna areas of Cameroon [23] |
|  |  | *Atolytus* | *A. agrestis* | This species was found in Maroua in the Logone and Chari division [23] |
|  |  |  | *A. fuscipes* | This species was identified in Garoua in the Sudan-savanna of North Cameroon [23] |
|  |  |  | *A. albipalpus* | This species was found in the Logone and Chari and Waza in the Sudan-savanna [23] |
|  |  | *Tabanus* | *Tabanus billingtoni* | This species was identified in Mt Cameroon and Bamoum [23] |
|  |  |  | **T. fasciatus* | This species was recently caught in the gallery forest in Galim of Ngaoundere. And in the Camp site, Oudou, Gabong and Minali sites of the SODEPA ranch of Ndokayo in the Mosaic-savanna |
|  |  |  | *T. marmorosus* | This species was identified around the Congo Basin and in Uganda. It can be found in Cameroon in areas such as Kumba, Lekie, Nyong and Mfoumou, Upper Sanaga, Ntem, Kribi, Mbam, Lom and Djerem. It was discovered in Garoua, Bertoua and Ngaoundere [23] |
|  |  |  | *T. obscurefumatus* | This is a rare species and was found in forest confinement and in transition zones like along the Kumba road and Ebolowa [23] |
|  |  |  | *T. sufis* | It was found in Logone and Chari [23]. It was recently identified in the Sora Mboum Sudan-savanna of North Cameroon by Lendzele et al. [33] |
|  |  |  | *T. pertinens* | This species was identified in Garoua in the Sudan-savanna of the North region of Cameroon [23] |
|  |  |  | *T. leucostostomus* | This species was identified in Maroua in the Sudan-savanna of Far North region of Cameroon [23] |
|  |  |  | *T. gratus* | It was found in Garoua and Logone and Chari [23]. It was recently caught in the savannas of Ngaoundere and North Cameroon [33] |
|  |  |  | *T. triquetrornatus* | This species was caught along river Nyong and seemed to be abundant in Cameroon [23] |
|  |  |  | *T. argenteus* | This species was found in Kumbo, Edea road and Bertoua [23]. It was recently found in the Douala-Edea Game Reserve |
|  |  |  | *T. variabilis* | This species was found in Lom and Djerem, Bertoua and Maroua [23] |
|  |  |  | *T. insiginis neavi* | This species was found in Kadei, Bertoua and frequent in mosaic-forest zones [23] |
|  |  |  | *T. canus* | This species was caught in Yaounde, Lekie and was noticed in the evening with light. It was caught in Maroua and usually found in forest and post forest areas [23] |
|  |  |  | *T. fulvicapillus* | This species was reported by Oldroyd [43] in Cameroon, but its locality was indicated in Congo Brazzaville |
|  |  |  | *T. rageani* | This species was found in Mefou, Kribi and Douala in forest relics [23] |
|  |  |  | *T. pluto* | This species was caught in Mamfe in the humid forest in the South West region of Cameroon [23] |
|  |  |  | *T. xanthomelas* | This species was found in Ivory Coast, Guinea, C.A.R and Cameroon (Benoue and river Fako) [23] |
|  |  |  | *T. biguttatus* | This species was found in Bertoua, Garoua, Maroua and Logone and Chari. It was recently identified in the North (Sora Mboum) and Ngaoundere |
|  |  |  | *T. anaeus* | This species was described around Kumba in the humid forest of the South West region [23] |
|  |  |  | *T. rufipes* | This species was signaled in all forest regions [23]. Recently caught in Minali locality of East region |
|  |  |  | *T. doaldsoni* | This species was caught in Esseka [23] |
|  |  |  | *T. scholae* | The series-type of this species was from Kumba in the humid forest of South West [23] |
|  |  |  | *T. par* | This species was identified in Mamfe, Maroua and Logone and Chari. This species was savanna-like and penetrated the forest. It was identified in the savanna of North Cameroon (Sora Mboum) by Lendzele et al. [33] |
|  |  |  | **T. latipes* | This species was recently caught at the palm oil plantation and around the abattoir in the Sanaga maritime zone |
|  |  |  | *T. zoulouensis* | This species was found in Bamoum in the west highland of West Cameroon [23] |
|  |  |  | *T. thoracinus* | This species was found in Maroua in the Sudan-savanna of the Far North region of Cameroon [23] |
|  |  |  | *T. boueti* | This species was caught in the Upper Nyong in the rain forest of Cameroon [23] |
|  |  |  | *T. besti* | Oldroyd [58] in Ovazza et al. [23] reported the occurrence of this species in Kumba, Ivory Coast, Foumbam and Lom and Djerem |
|  |  |  | *T. ianthinus* | This species was identified in Nyong and Mfoumou and Mbarain in the Mosaic-forest of Cameroon [23] |
|  |  |  | *T. lubutuensis* | This species was caught in Fako, Meme, Mefou and Lekie. It was confined in the forest relics of Cameroon [23] |
|  |  |  | *T. obscurehirtus* | This species was identified to be widespread in all forest zones [23] |
|  |  |  | *T. combustus* | This species was known by Oldroyd to be found in the forest. During this period, it was not signalled in Cameroon. It was not equally signalled in present collections |
|  |  |  | *T. secedens* | This species was caught in all forest and post forest regions such as Mefou, Lekie, Nyong and Soo and Nyong and Mfoumou [23]. It was recently caught in the Diddel tanne site of the Far North Cameroon |
|  |  |  | *T. congoiensis* | This species was widespread in Cameroon especially in areas such as Lekie, Nyong and Soo, Nyong and Mfoumou, Upper Sanaga, Upper Nyong, Ebolowa and Bertoua [23] |
|  |  |  | *T. crosskeyi* | This species was described in Guinea (Bisao). Series of females were captured in the forest of Cameroon (Ekombitie) [23] |
|  |  |  | *T. conformis* | This species was collected along the slope of Mt Cameroon, river Mungo and Foumbam [23] |
|  |  |  | *T. fuscipleuris* | This species was collected in Manyu in the humid forest of the South West region of Cameroon [23] |
|  |  |  | *T. taeniola* | This species existed in all parts of Cameroon and was abundant in savanna regions. It occurred in Manyu, Garoua, Nyong and Mfoumou, Ayos and Upper Nyong [23]. It was recently identified in the savanna of North Cameroon [33] |
|  |  |  | *T. leverani* | This species was present in Cameroon, but abundant in Guinean-savanna zones [23] |
|  |  |  | *T. hamoni* | This species was described in the Upper Volta of Nigeria, C.A.R., Logone and Chari and Waza [23] |
|  |  |  | *T. nyasac* | This species was identified in the Logone and Chari division of the Far North of Cameroon [23] |
|  |  |  | **T. ricardae* | This species was recently caught in the Guinee-savanna notably in Velambai and Vina du Sud |
|  |  |  | *T. ustus* | This species was reported by Brygoo to occur in the region of Ayos |
|  |  | *Hybromitra* | *Hybromitra severini* | This species was found in Mefou, 50 km along the Yaounde-Ebolowa road and in Nkolbissong [23] |
| Pangoniinae | Philolichini | *Philolichie* | *Philolichie semilivida* | This species was reported in Kumba, Mefou, Mbalmayo and Kribi [23] |
|  |  |  | *P. rodhaini* | Oldroyd [44] described this species as *Stenophara adama* and Rageau et al. [21] described it in Cameroon in the Lekie |
|  |  |  | *P. gravoti* | This species was identified in Mefou, Nyong and Soo, Ntem and Ebolowa [23] |
| Chrysopinae | Chrysopini | *Chrysops* | *C. zahrai* | This species was found in Donga Mantung, Oku (altitude of 2000 m), summit savannas and Ngaoundere road (altitude of 1300-1500 m) [23] |
|  |  |  | *C. griseicollis* | This species was described in Kumba in the humid forest of the South West region of Cameroon [23] |
|  |  |  | *C. maximus* | This species was caught in Kumba, Ndop plain (marshy savanna and forest areas at altitude of 1100 m). It was also caught in Foumbam at altitude of 1300 m in savanna and forest-galleries [23] |
|  |  |  | *C. dimidiata* | This species was signalled in Kumba and Oku forest [23]. It was identified around the Douala-Edea Game reserve |
|  |  |  | *C. silacea* | This species was signalled in Nigeria. *C. dimidiata* and *C. silacea* coexisted in the same habitat. It was also reported in Congo Brazzaville by Ovazza and Taufflier [59]. Recently identified around the Douala-Edea wildlife area |
|  |  |  | *C. langi* | This species was caught in Kumba, Mefou and Yabassi [23] |
|  |  |  | *C. longicornis* | This species was found in Manyu, Kribi, Ambam and Wouri. It was recently identified in the Adamawa Plateau |
|  |  |  | *C. funebris* | This species was found in Meme and Mezam by Ovazza et al. [23] and recently signalled in Oudou and Gabong sites of the mosaic-forest of East Cameroon |
|  |  |  | **C. distinctipennis* | This species was recently identified by Lendzele et al. [33] in Sahel savanna of North Cameroon. It was formerly described in the document of Ovazza et al. [23] as *C. stigmaticalis* |
|  | Rhinomyzini | *Sphecodemyia* | *Sphecodemyia gromieri* | This species was caught in Kribi in the rainforest [23] |
|  |  | *Thriambeutes* | *T. nigripennis* | This species was collected at River Ngoko [23] |
|  |  | *Jashinea* | *Jashinea praestabilis* | This species was collected in Kumba in the humid forest of the South West region of Cameroon [23] |
|  |  |  | *Jashinea rodhaini* | This species was collected from Mefou [23] |
|  |  | *Tabanocella* | *T. stimulans* | This species was collected from Kumba, Nyong and Mfoumou, Lekie, Nyong and Soo, Ntem and Kribi [23] |
|  |  |  | *T. schoutedeni* | This species was very rare but was found along the Bamenda-Mamfe road [23] |
|  |  |  | *T. oldroydi* | This species was collected from Lekie and Mefou [23] |
|  |  | *Thaumastocera* | *T. akwa* | The series-type of this species was collected from Lolodorf and Kumba in Cameroon [23] |
|  | Bouvieromyini | *Mesomyia* | *Mesomyia apiformis* | This species was collected along the river Sanaga area and livestock areas of Kumba [23] |

**References**

1. Oldroyd H. The Horse-flies of the Ethiopian Region (Diptera: Tabanidae). 1. *Haematopota* and *Hippocentrum*. British Museum (Natural History), London, UK; 1952.p.226.
2. Ovazza M, Taufflieb R. Tabanides d’A.E.F. Bzzll. Z.E.C., n.s.1952;4:131–141.
